# The effects of long-term resistance exercise on the relationship between neurocognitive performance and GH, IGF-1, and homocysteine levels in the elderly

**DOI:** 10.3389/fnbeh.2015.00023

**Published:** 2015-02-10

**Authors:** Chia-Liang Tsai, Chun-Hao Wang, Chien-Yu Pan, Fu-Chen Chen

**Affiliations:** ^1^Lab of Cognitive Neurophysiology, Institute of Physical Education, Health and Leisure Studies, National Cheng Kung UniversityTainan, Taiwan; ^2^Department of Physical Education, National Kaohsiung Normal UniversityTainan, Taiwan; ^3^Department of Recreation Sport and Health Promotion, National Pingtung University of Science and TechnologyPingtung, Taiwan

**Keywords:** resistance exercise, aging, cognition, IGF-1, GH, homocysteine, neuroelectric

## Abstract

This study aimed to investigate the effects of a long-term resistance exercise intervention on executive functions in healthy elderly males, and to further understand the potential neurophysiological mechanisms mediating the changes. The study assessed forty-eight healthy elderly males randomly assigned to exercise (*n* = 24) or control (*n* = 24) groups. The assessment included neuropsychological and neuroelectric measures during a variant of the oddball task paradigm, as well as growth hormone (GH), insulin-like growth factor-1 (IGF-1), and homocysteine levels at baseline and after either a 12 month intervention of resistance exercise training or control period. The results showed that the control group had a significantly lower accuracy rate and smaller P3a and P3b amplitudes in the oddball condition after 12 months. The exercise group exhibited improved reaction times (RTs), sustained P3a and P3b amplitudes, increased levels of serum IGF-1, and decreased levels of serum homocysteine. The changes in IGF-1 levels were significantly correlated with the changes in RT and P3b amplitude of the oddball condition in the exercise group. In conclusion, significantly enhanced serum IGF-1 levels after 12 months of resistance exercise were inversely correlated with neurocognitive decline in the elderly. These findings suggest that regular resistance exercise might be a promising strategy to attenuate the trajectory of cognitive aging in healthy elderly individuals, possibly mediated by IGF-1.

## Introduction

While life expectancy has been increasing in developed countries, one risk associated with a rapid growth in aged populations is that the number of people suffering from cognitive impairments and dementia could increase, due to age-related deteriorations in an array of cognitive processes involving central executive functions, attention, and short- and long-term memory (Anderson and McConnell, 2007). This can be attributed to gradual declines in physical activity levels (Kimura et al., 2013), the structural integrity of the brain (e.g., frontal, parietal, and temporal lobes) (Jernigan et al., 2001; Raz et al., 2004), and the secretion of growth factors (e.g., growth hormone (GH) and insulin-like growth factor-1 (IGF-1)) from the neurochemical system (Sonntag et al., 2005), which inevitably occur with aging. Cognitive impairment is closely related to decreases in both living-independence and general health. Therefore, determining how to counteract neurocognitive decline in order to reduce the costs associated with geriatric care is becoming an important issue for many public health systems around the world.

Recently, researchers have focused on the role of the GH/IGF-1 axis in attenuating age-related neurocognitive decline. Elderly individuals experience a fall in the ability to secrete GH, and a subsequent decrease in its secondary mediator (i.e., IGF-1), which is primarily produced in the liver (90%) but also by other cell types in the brain and vasculature (Sonntag et al., 2005). Both substances cross the blood-brain barrier and bind to receptors in the central nervous system to stimulate the growth of glial cells, myelination, and neurons: therefore, age-related decreases in serum GH and IGF-1 levels might contribute to cognitive decline in the elderly (Sonntag et al., 2005). A number of studies have attempted to understand the relationship between neuropsychological test performance and serum GH and IGF-1 levels in the elderly. Although some argue that there is no relationship between serum IGF-1 and GH concentrations and specific neurocognitive functions (e.g., GH vs. event-related potential (ERP) N2b component; IGF-1 vs. delayed recall and Brus reading) (Papadakis et al., 1995; Aleman et al., 1999; Quik et al., 2012), there is growing evidence for a significant association between neuropsychological measures (e.g., GH vs. reaction times (RTs) in selective attention and short-term memory; IGF-1 vs. speed of information processing) and serum IGF-1 and GH levels in elderly populations (Rollero et al., 1998; Aleman et al., 1999; Kalmijn et al., 2000; Dik et al., 2003; Quik et al., 2012). These cross-sectional studies, however, do not provide sufficient evidence for effective interventions to prevent or reduce the risk of cognitive decline in the elderly. The inconclusive findings of such studies regarding the relationship between serum GH and IGF-1 levels and age-related neurocognitive decline in healthy elderly individuals mean that more research needs to be carried out that uses long-term exercise interventions to clarify the role of growth factors in this phenomenon.

Exercise is a lifestyle factor crucial to the prevention or delayed onset of mild cognitive impairment in later life (Smith et al., 2013). The potential mechanisms for this effect include exercise-induced increases in the levels of nerve growth factors, such as GH and IGF-1, as these play central roles in the health of neurons in the brain. Previous studies investigating the effect of resistance training on cognition mostly discussed the potential mechanisms using GH, IGF-1, and homocysteine, possibly due to the fact that these biomarker secretions are exercise-sensitive, since they are characterized by different physiological and metabolic demands (Cassilhas et al., 2007, 2010; Seo et al., 2010). However, several experimental studies suggest that basal levels of serum IGF-1 (Singh et al., 1999; Cassilhas et al., 2007, 2010) and GH (Seo et al., 2010) in the elderly are only affected by long-term resistance training at moderate and high intensities.

A growing number of studies strongly support the beneficial effects of resistance exercise on various aspects of cognitive performance, such as on executive control, memory, attention, and mini mental state examination (MMSE) score (Perrig-Chiello et al., 1998; Ozkaya et al., 2005; Cassilhas et al., 2007; Liu-Ambrose et al., 2010). In contrast, Kimura et al. (2013) and Venturelli et al. (2010) failed to replicate these results, reporting that 3 months of resistance training did not significantly improve cognitive performance (e.g., on MMSE and task switching) in healthy elderly populations. These inconsistent results can be attributed to differences in the forms of resistance exercise that were prescribed, and the neuropsychological measures that were assessed. The kinds of resistance exercise that successfully enhanced cognition in elderly subjects included moderate to high intensity training performed two or three times per week. Additionally, different cognitive functions require different training periods: for example, short-term (e.g., 8 weeks) resistance training can only benefit short-term memory (Perrig-Chiello et al., 1998), whereas long-term (e.g., 12 months) resistance training is better with regard to enhancing long-term memory and executive functions (Liu-Ambrose et al., 2010).

Executive functions are susceptible to senescence (Alvarez and Emory, 2006), as the neural networks involved in these are subject to age-related atrophy (West et al., 2010; O’Connell et al., 2012). The oddball task is a cognitive test extensively used by previous studies to examine the effects of aging on executive functions involving information processing. The performance of ERPs during the oddball task is a sensitive index of the changes in neural activity related to cognition that are associated with aging, with the P300s (e.g., P3a and P3b) being the most sensitive biomarkers for normal aging and age-related pathology among the neuroelectric markers elicited during this task (Pontifex et al., 2009; West et al., 2010; O’Connell et al., 2012). Since neuronal loss in the cerebral cortex and the cerebral white matter commences during the third decade of life (Jernigan et al., 2001), this causes a linear decrease in the amplitude of the potentials, coupled with a marked anterior shift in the topographical orientation of the P300 components observed in the elderly when performing the oddball task (West et al., 2010; Richardson et al., 2011; O’Connell et al., 2012). Fortunately, executive functions are more strongly affected by physical activity or exercise than other aspects of cognitive functioning (Colcombe and Kramer, 2003). A number of studies have demonstrated that active lifestyles in the elderly can enhance performance on the oddball task. For example, fitter elderly subjects showed shorter RTs and larger P3b amplitudes compared with less-fit age-matched counterparts (Pontifex et al., 2009). Similarly, physically active elderly subjects with habitual moderate exercise demonstrated better performance on the oddball task, such as faster RTs and larger P3 amplitudes (McDowell et al., 2003; Hatta et al., 2005). Exercise intervention could thus elicit training effects on the performance of such cognitive tasks in the elderly.

Previous studies on exercise and brain aging have intensively examined neuropsychological (i.e., behavioral and psychomotor) measures. However, thus far, research has not yet considered the effects of long-term resistance exercise on neuroelectric performance, nor has it explored the potential neurophysiological mechanisms impacting performance of the visual oddball task in healthy elderly individuals. Therefore, the aims of this study were as follows: (1) to investigate the effects of a 12 month resistance exercise intervention on executive functions, with respect to age-related effects on neuropsychological and neuroelectric performance elicited during the visual oddball task; and (2) to further explore whether changes in basal levels of serum GH, IGF-1, and homocysteine relate to the effects of long-term resistance exercise on executive functions. Based on the above review of research on cognition and neurobiology, the current study examined the following hypotheses: (1) long-term resistance exercise can attenuate or minimize certain aspects of the progression of cognitive degeneration in healthy elderly individuals when performing an oddball task designed to elicit P3a and P3b components; and (2) exercise-induced changes in growth factors can act as potential mechanisms for this effect. We believe the combination of neuropsychological and neuroelectric measures, with biochemical measures obtained during long-term exercise intervention, can provide more compelling evidence and deeper insights into the nature of the effects of exercise on attenuation or prevention of the cognitive decline associated with aging.

## Methods

### Subjects

This study recruited forty-eight elderly males (aged 65–79 years, mean 71.40 ± 3.79) from senior community centers in rural areas of Taiwan, with these subjects being more sedentary and less educated than their city-dwelling counterparts, and thus presumably more responsive to exercise training. We only recruited male subjects because the effects of exercise on endocrinological responses may be gender-dependent (Baker et al., 2010). Subjects were recruited with the use of print advertisements and underwent screening by a standardized telephone interview. They then underwent a medical examination, including heart rate and blood pressure measurements, electrocardiography, routine laboratory testing, a standardized neurological examination, and a structured interview on their previous medical histories. The medical exam ascertained whether subjects were free of a history of brain injury, liver and kidney dysfunction, severe medical conditions affecting the dopaminergic system, neurological disorders, and psychiatric illnesses, such as depressive symptoms [defined by scores above 13 on the Beck Depression Inventory, second edition (BDI-II)], dementia, and mild cognitive impairment (defined by scores below 26 on the MMSE) (Ruscheweyh et al., 2011). The Edinburgh Handedness Inventory assessed all subjects as right-handed (Oldfield, 1971). The study obtained written informed consent from all participants, and was approved by the Institutional Ethics Committee of National Cheng Kung University.

### Study procedure

**Figure 1:**
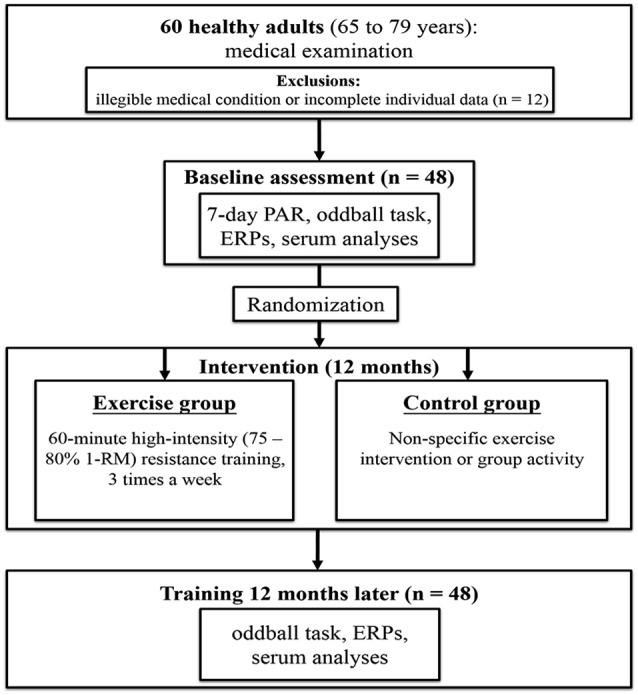
**The CONSORT (Consolidated Standards of Reporting Trials) flow chart**.

Figure 1 presents the procedure of this study. The original cohort consisted of 60 subjects. Based on the assessment of two physicians specializing in geriatric care and physiotherapy, three subjects were excluded due to incomplete individual data, three due to high blood pressure, and six due to musculoskeletal problems, neurological disorders, or psychiatric illness (e.g., scores above 13 on the BDI-II or below 26 on the MMSE), leaving a total of 48 subjects for the present study. Participants also completed the 7-day physical activity recall questionnaire (7-day PAR; Sallis et al., 1985) to ascertain their previous levels of physical activity, in order to provide sufficient pre-activity screening to lower potential risk factors prior to long-term resistance training. The 48 participants were randomized to the exercise group (i.e., resistance exercise intervention) or control group after matching for age and baseline level of physical activity. The two groups did not significantly differ at baseline in any of the demographic characteristics, including years of formal education, body mass index, years of smoking, MMSE, BDI-II, or systolic and diastolic pressure (see Table 1).

**Table 1:** **Demographic characteristics of the exercise and control groups**.

	Exercise group (*n* = 24)	Control group (*n* = 24)	*p*
Age (years)	70.79 ± 3.39	72.00 ± 4.14	0.274
Body mass index (kg/m^2^)	25.96 ± 2.50	24.63 ± 3.60	0.144
Education (years)	6.63 ± 2.50	6.75 ± 2.71	0.869
Smoking (years)	7.42 ± 10.80	6.33 ± 12.52	0.750
Systolic pressure (mmHg)	141.75 ± 15.21	138.38 ± 13.28	0.417
Diastolic pressure (mmHg)	83.83 ± 7.40	84.96 ± 6.32	0.514
MMSE	27.88 ± 1.19	28.21 ± 0.98	0.295
BDI-II	5.63 ± 1.76	5.38 ± 2.30	0.675
7-day PAR (Kcal/d)	1776.63 ± 400.29	1841.38 ± 431.15	0.592
Changes in strength (post-pre)
Biceps curls	9.25 ± 6.87	−1.38 ± 1.56	<0.001
Triceps extensions	6.38 ± 3.90	−0.96 ± 1.94	<0.001
Seated rowing	16.29 ± 6.12	−2.71 ± 4.02	<0.001
Hamstring curls	8.67 ± 7.14	−1.67 ± 3.17	<0.001
Latissimus dorsi pull-downs	15.38 ± 8.61	1.08 ± 2.93	<0.001
Calf raises	7.08 ± 4.13	0.63 ± 2.89	<0.001
Leg press	22.96 ± 7.95	−0.63 ± 4.98	<0.001

*MMSE, mini mental state examination; BDI, Beck Depression Inventory; 7-day PAR,7-day physical activity recall; 1RM: one-repetition maximum*.

Two certified fitness instructors completed all assessments of one repetition maximum (1-RM) and peak muscle power for each participant within 1 week of the completion of the baseline evaluation. All the participants in the exercise group were familiarized with the use of free weights and bodybuilding machines before the formal resistance exercise program. On a separate day during the week after the baseline evaluation, the subjects had blood withdrawn between 8:30 and 9:30 AM following overnight fasting, and then performed a cognitive task test with concomitant neuroelectric recording (i.e., ERPs).

After 12 months, the participants completed the same questionnaires, had blood withdrawn, and received measures of neurocognitive parameters over a period of 1 week.

### Cognitive task and experimental procedure

A laptop computer monitor displayed all white stimuli, including oddball stimuli, standard stimuli, and novel stimuli, against a black background. Pontifex et al. (2009) suggested that the three-stimulus oddball task has a higher level of cognitive difficulty with regard to stimulus discrimination among the elderly. The oddball stimulus was the geometric figure “○”, and the standard stimulus was the geometric figure “□”. The novel stimulus category consisted of the following 10 figures: ⋇, ＊, ⊚, ◇, △, ▽, ⨀, ⊕, ♀, ♂, ⊲. The center of the computer screen (width = 43 cm), located directly in front of the participant at face level at a distance of approximately 75 cm, displayed the stimulus (4.09° × 4.09°).

The cognitive test (oddball task) paradigm presented the three types of stimuli in different proportions, with 20% oddball, 60% standard, and 20% novel stimuli. The monitor presented each stimulus for 500 ms, followed by a blank screen for 1500 ms. The stimulus would disappear immediately after the participants responded. If the participants did not respond within 2000 ms, the stimulus would disappear, and the program would advance to the next trial. Participants pressed the “M” key in response to the oddball stimulus, and the “B” key in response to the standard or a novel stimulus. Stimuli were presented in a different, random order for each participant. The entire experiment consisted of three blocks of 100 trials, with the order of the stimulus blocks counterbalanced across participants. Participants were instructed to respond as quickly and accurately as possible. The present study adapted the oddball task from West et al. (2010), wherein its capacity to effectively differentiate lower executive functions of older adults from younger adults was demonstrated.

A trained experimenter blind to group assignment performed the cognitive testing. The experiment was administered in an acoustically shielded room with dimmed lights. On arrival at the laboratory, the experimenter explained the procedure and made sure that the participants were familiar with it. Participants completed 30 practice trials prior to the formal test to ensure they understood the whole process. The electrocap and electro-oculographic (EOG) electrodes were attached to the head and face of the participants before the formal test. Each participant was asked to sit comfortably in an adjustable chair in front of a laptop computer display driven by an IBM-compatible personal computer with a stimulation system (Neuroscan Ltd., EI Paso, USA). During the test, the experimenter sat next to the participant to monitor visual fixation. The experimenter gave verbal encouragement to look at the screen if they detected the participant’s eyes moving away from the central stimulus during the execution of a response. All participants with normal or corrected-to-normal vision acuity performed the oddball task with simultaneous recording of ERPs.

### Electrophysiological recording and analysis

Electroencephalographic (EEG) activity was recorded from 18 electrode sites (F7, F8, F3, F4, Fz, T3, T4, C3, C4, Cz, T5, T6, P3, P4, Pz, O1, O2, and Oz), using an elastic electrode cap (Quik-Cap, Compumedics Neuroscan, Inc., El Paso, TX) designed for the International 10–20 System. Additional ocular electrodes placed on the supero-lateral right canthus and infero-lateral to the left eye monitored horizontal and vertical EOG (i.e., HEOG and VEOG) activity for eye movements. Scalp locations were referred to linked mastoid electrodes, while a ground electrode was placed on the mid-forehead on the Quik-Cap. All electrode impedances were below 5 kΩ. EEG data acquisition employed an A/D rate of 500 Hz/channel, a band-pass filter of 0.1–50 Hz, and a 60 Hz notch filter, with continuous writing to hard disk for off-line analysis using SCAN4.3 analysis software (Compumedics Neuroscan, Inc., El Paso, USA).

The ERP analysis epochs extracted off-line consisted of segments from −100 ms of pre-stimulus activity to 1000 ms of post-stimulus activity. Trials with a response error or EEG artifacts (e.g., VEOG, HEOG, and electromyogram) exceeding peak-to-peak deflections over 100µV were rejected before averaging. The remaining effective data was assembled according to the three different conditions (i.e., oddball, standard, and novel). Measures of peak amplitude were calculated for two components to quantify the effects of long-term exercise intervention and stimulus type on the ERPs. Since P3a has a more anterior distribution than P3b (O’Connell et al., 2012), West et al. (2010) outlined the following definitions for P3 amplitudes: the novelty P3 amplitude (i.e., P3a) is the major positive deflection over the anterior scalp (F3 and F4), and the most positive point between 300 ms and 400 ms after the stimulus. In contrast, the P3b amplitude is the major positive deflection over the central and posterior scalps (Cz, Pz, and Oz), and the most positive point between 300 ms and 800 ms.

### Resistance training prescription

Resistance training classes for the exercise group began within 1 week after initial 1-RM testing. Two certified fitness instructors formally trained and educated by professional physical fitness courses led the classes at a university fitness center. The exercise group was divided into small subgroups of three to six participants. Each training class lasted approximately 60 min, with 10 min of warm-up, 40 min of core content, and 10 min of cool-down. The warm-up included slow-paced walking and active mobility exercises for the joints of the four limbs. The core resistance exercise content implemented a circuit-training schedule with a progressive, high-intensity protocol (Liu-Ambrose et al., 2010). The training circuit consisted of the following exercises in the order stated: biceps curls, leg presses, triceps extensions, hamstring curls, latissimus dorsi pull-downs, calf raises, and seated rowing. The training equipment included bodybuilding machines and free weights. The participants performed the resistance training at an intensity of 75–80% 1-RM for three sets of 10 repetitions, at an average speed, with a 90-second rest between sets, and a 3 min interval between each apparatus. The load pressed or lifted for each exercise was recorded in each participant’s exercise log at every class. As each individual’s muscle strength increased, their prescribed training load was also raised to ensure they performed the training at intensity levels corresponding to 75–80% of 1-RM. Such an intensity protocol led to increases in serum IGF-1 levels in elderly subjects in previous studies (Singh et al., 1999; Cassilhas et al., 2007, 2010). During the cool-down period, participants used a variety of relaxation techniques, such as controlled breathing and static stretching exercises (i.e., maintaining maximal muscle elongation for 30s to increase range of motion). The fitness instructors recorded adherence, expressed as the percentage of classes attended, in the participants’ exercise logs. The attendance rate was above 90% for all participants, with no subjects dropping out of the study.

The participants in the exercise group were required to participate in 60 min resistance training classes three times a week for a period of 12 months. Participants in the control group received baseline and post-intervention evaluations, but did not receive a specific intervention or group activity that would prevent any potential cognitive benefits from social interactions they might have engaged in.

### Serum analysis

Prior to the two cognitive task tests, a trained phlebotomist withdrew blood from the antecubital vein using an aseptic technique for analysis of serum IGF-1, GH, and homocysteine. The blood was allowed to clot (BD Vacutainer Plus), and then centrifuged at 3000 rpm for 15 min at 4°C (Hettich Mikro 22R, C1110). Each sample was frozen and stored at –80°C for further serum marker assays. Serum values of GH, IGF-I, and homocysteine were determined by a chemiluminescence immunoassay method using an Access Ultrasensitive hGH reagent pack (Beckman Coulter Inc, USA), Liaison IGF-1 reagent (DiaSorin S.P.A., Italy), and Siemens reagents for homocysteine assay (Siemens Healthcare Diagnostics Inc., USA), respectively. The detection limit for GH was 0.002 ng/mL, while that for the IGF-1 was 3 ng/mL, and that for homocysteine was 0.50 mmol/L. All the procedures to assess GH, IGF-I, and homocysteine were performed by the same person to avoid inter-operator bias.

### Statistical analysis

Independent *t*-tests were used to examine the homogeneity of the demographic backgrounds of the subjects in the exercise and control groups. The accuracy rates and correct-trial RTs were submitted separately to a 2 (*Group*: exercise vs. control) × 2 (*Time*: pre-exercise vs. post-exercise) × 3 (*Condition*: oddball vs. standard vs. novel) mixed repeated measures analysis of variance (RM–ANOVA). P3 amplitudes from ERP recordings were submitted separately to a 2 (*Group*: exercise vs. control) × 2 (*Time*: pre-exercise vs. post-exercise) × 3 (*Condition*: oddball vs. standard vs. novel) × 2 (*Electrode*: F3 vs. F4 for the P3a component; Cz vs. Pz vs. Oz for the P3b component) RM–ANOVA. All biochemical markers were submitted separately to a 2 (*Group*: exercise vs. control) × 2 (*Time*: pre-exercise vs. post-exercise) RM–ANOVA. Appropriate multiple comparisons were performed following any simple main effects. When a significant difference occurred, Bonferroni *post hoc* analyses were performed. The Greenhouse–Geisser (G–G) correction adjusted the significance levels of the *F* ratios whenever RM–ANOVA detected a major violation of the sphericity assumption. Partial Eta squared ($\eta_{P}^{2}$) was used to calculate effect sizes for significant main effects and interactions, with the following standards used to determine the magnitude of mean effect size: 0.01–0.059 represented a small effect size; 0.06–0.139, a medium effect size; and >0.14, a large effect size. Pearson product–moment correlations were used to examine changes in the biochemical markers and cognitive variables. Significance was set at *p* < 0.05 for all analyses.

## Results

### Accuracy rate

**Figure 2:**
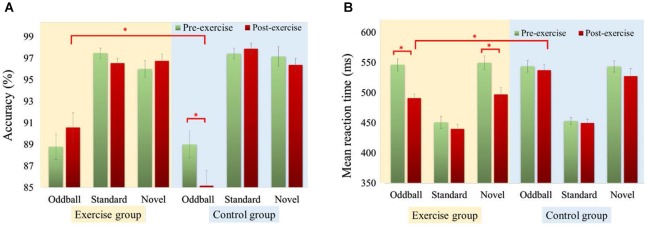
**Accuracy rate (A) and reaction time (B) performance of exercise and control groups during the oddball task at baseline and 12 months later in three conditions**. *Significantly different (*p* < 0.05). Statistical values expressed as mean ± SE.

RM–ANOVA performed on the accuracy rates (see Figure 2A) highlighted a main effect of *Condition* [*F*_(2,92)_ = 102.72, *p* < 0.001, $\eta_{P}^{2}$ = 0.69], with a lower accuracy rate for oddball (88.4%) than standard (97.3%) and novel (96.6%) conditions. The interactions between *Time* × *Group* [*F*_(1,46)_ = 5.34, *p* = 0.025, $\eta_{P}^{2}$ = 0.10], *Condition* × *Group* [*F*_(2,92)_ = 3.42, *p* = 0.037, $\eta_{P}^{2}$ = 0.07], and *Time × Condition × Group* [*F*_(2,92)_ = 5.39, *p* = 0.006, $\eta_{P}^{2}$ = 0.11] were also significant. *Post hoc* analysis showed a lower accuracy rate for the oddball condition in the control group 12 months after baseline [*t*_(23)_ = 3.14, *p* = 0.005]. The exercise group exhibited a significantly higher accuracy rate in the oddball condition [*t*_(46)_ = 2.77, *p* = 0.008] compared to the control group after 12 months.

### Reaction time

As shown in Figure 2B, RM–ANOVA conducted on mean RTs revealed a main effect of *Time* [*F*_(1,46)_ = 26.47, *p* < 0.001, $\eta_{P}^{2}$ = 0.37], and a main effect of *Condition* [*F*_(2,92)_ = 210.94, *p* < 0.001, $\eta_{P}^{2}$ = 0.82], suggesting that RTs were faster after the exercise intervention (490.51 ms) than before it (514.52 ms), and that RTs were faster in the standard condition (448.35 ms) than in both the oddball (529.67 ms) and novel (529.52 ms) conditions. The interactions of *Time × Group* [*F*_(1,46)_ = 10.91, *p* = 0.002, $\eta_{P}^{2}$ = 0.19], *Time* × *Condition* [*F*_(2,92)_ = 8.35, *p* < 0.001, $\eta_{P}^{2}$ = 0.15], and *Time* × *Condition* × *Group* [*F*_(2,92)_ = 4.22, *p* = 0.018, $\eta_{P}^{2}$ = 0.08] were also significant. *Post hoc* analysis showed the exercise group responded faster in the oddball [*t*_(23)_ = 5.10, *p* < 0.001] and novel [*t*_(23)_ = 5.27, *p* < 0.001] conditions after exercise intervention compared to baseline. The exercise group only showed significantly faster responses than the control group in the oddball condition [*t*_(46)_ = −3.97, *p* < 0.001] after 12 months.

### P3a amplitude

**Figure 3:**
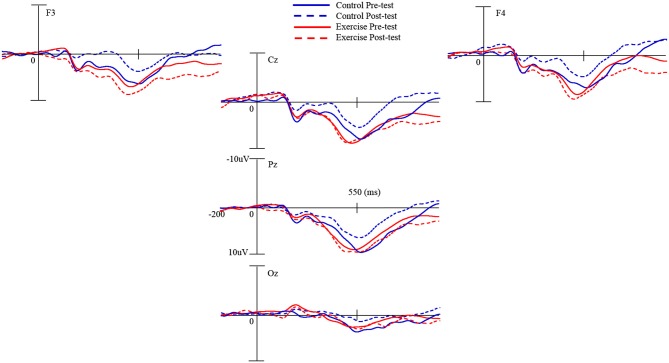
**Grand average event-related potential waveforms of five electrodes (F3 and F4 for the P3a component; Cz, Pz, and Oz for the P3b component) in the oddball condition during pre- and post-tests of exercise and control groups**.

As illustrated in Figure 3, RM–ANOVA performed on the P3a amplitudes showed a main effect of *Condition* [*F*_(2,92)_ = 7.66, *p* = 0.001, $\eta_{P}^{2}$ = 0.14], and a main effect of *Electrode* [*F*_(1,46)_ = 14.64, *p* < 0.001, $\eta_{P}^{2}$ = 0.24], suggesting that the P3a amplitude was significantly smaller in the standard condition than in the novel condition, and significantly larger for the F4 electrode than for the F3 electrode. The interactions of *Time* × *Condition* [*F*_(2,92)_ = 3.29, *p* = 0.042, $\eta_{P}^{2}$ = 0.07], *Condition × Electrode* [*F*_(2,92)_ = 7.54, *p* = 0.001, $\eta_{P}^{2}$ = 0.14], *Time* × *Condition* × *Electrode* [*F*_(2,92)_ = 3.90, *p* = 0.024, $\eta_{P}^{2}$ = 0.08], and *Time* × *Condition* × *Group* [*F*_(2,92)_ = 3.49, *p* = 0.034, $\eta_{P}^{2}$ = 0.07] were also significant. *Post hoc* analysis showed that only the P3a amplitude in the oddball condition [*t*_(23)_ = 3.19, *p* = 0.004] was significantly smaller across all electrodes in the control group after 12 months. The exercise group exhibited significantly larger P3a amplitudes in the oddball condition [*t*_(46)_ = 2.40, *p* = 0.001] across all electrodes compared to the control group after 12 months.

### P3b amplitude

RM–ANOVA performed on the P3b amplitudes showed a main effect of *Condition* [*F*_(2,92)_ = 4.07, *p* = 0.020, $\eta_{P}^{2}$ = 0.08], and a main effect of *Electrode* [*F*_(2,92)_ = 153.23, *p* < 0.001, $\eta_{P}^{2}$ = 0.77], suggesting that the P3b amplitude was significantly larger in the oddball condition than in the standard condition, and significantly smaller for the Oz electrode than for the Cz and Pz electrodes. The interactions of *Time* × *Condition* [*F*_(2,92)_ = 4.03, *p* = 0.021, $\eta_{P}^{2}$ = 0.08], *Condition × Electrode* [*F*_(4,184)_ = 15.61, *p* < 0.001, $\eta_{P}^{2}$ = 0.25], *Condition* × *Electrode* × *Group* [*F*_(4,184)_ = 2.98, *p* = 0.021, $\eta_{P}^{2}$ = 0.06], and *Time* × *Condition* × *Group* [*F*_(2,92)_ = 3.35, *p* = 0.039, $\eta_{P}^{2}$ = 0.07] were also significant. *Post hoc* analysis showed that only P3b amplitude in the oddball condition [*t*_(23)_ = 3.61, *p* = 0.001] was significantly smaller across all electrodes in the control group after 12 months. The P3b amplitude approached significance [*t*_(46)_ = 2.02, *p* = 0.050] between the two groups in the oddball condition across all electrodes after 12 months.

### GH

**Figure 4:**
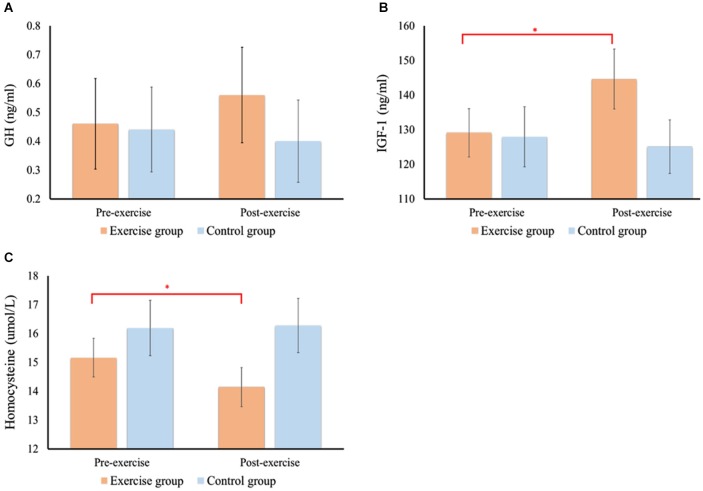
**Changes in serum levels of GH (A), IGF-1 (B), and homocysteine (C) before and after 12 months in the exercise or control group**. *Significantly different (*p* < 0.05) from corresponding baseline values. Statistical values expressed as mean ± SE.

Figure 4A shows the levels of all biochemical markers before the intervention and after 12 months in the exercise and control groups. RM–ANOVA performed on serum GH levels showed that neither a significant main effect of *Group* or *Time* nor a significant interaction of *Time* × *Group* was present, indicating that the training effect of serum GH levels did not differ between two groups.

### IGF-1

RM–ANOVA performed on serum IGF-1 levels (see Figure 4B) showed a main effect of *Time* [*F*_(1,46)_ = 5.33, *p* = 0.025, $\eta_{P}^{2}$ = 0.10], and the interaction of *Time* × *Group* [*F*_(1,46)_ = 11.24, *p* = 0.002, $\eta_{P}^{2}$ = 0.20] to be significant. *Post hoc* analysis showed that serum IGF-1 levels significantly increased in the exercise group after 12 months of resistance exercise. The changes in IGF-1 levels in the exercise group were significantly correlated with the changes in RTs (*r* = −0.47, *p* = 0.020) and P3b amplitude (*r* = 0.52, *p* = 0.009) in the oddball condition. However, this effect was not found for the accuracy rates (*r* = 0.17, *p* = 0.939) or P3a amplitudes (*r* = 0.32, *p* = 0.123).

### Homocysteine

As can be seen from Figure 4C, RM–ANOVA performed on serum homocysteine levels showed *Time* [*F*_(1,46)_ = 9.49, *p* = 0.003, $\eta_{P}^{2}$ = 0.71], and the interaction of *Time* × *Group* [*F*_(1,46)_ = 4.85, *p* = 0.033, $\eta_{P}^{2}$ = 0.10], to produce significant main effects. *Post hoc* analysis showed that serum homocysteine levels were only significantly reduced in the exercise group after 12 months of resistance exercise. However, there were no significant correlations between changes in homocysteine levels and changes in behavioral and ERPs performances after long-term intervention in the exercise group.

## Discussion

This study aimed to investigate whether a 12 month high-intensity resistance exercise intervention could effectively retard a decline in executive functions in healthy elderly males, and to determine the relationship between changes in IGF-1, GH and homocysteine levels and neurocognitive performance (e.g., neuropsychological and neuroelectric components) during an oddball task. The control group displayed a lower accuracy rate and smaller P3a and P3b amplitudes in the oddball condition when performing the oddball task after 12 months. The results for the exercise group showed that long-term high-intensity resistance exercise can decrease RTs and attenuate decreases in P3a and P3b amplitudes during a stimulus discrimination task, as well as increase serum IGF-1 levels and decrease serum homocysteine levels, and that changes in IGF-1 levels were significantly correlated with changes in RTs and P3b amplitudes in the oddball condition. These findings suggest that long-term resistance exercise could be an effective mechanism for attenuating the age-related decreases in neural efficiency in healthy elderly individuals manifested during the oddball task, possibly modulated by increased IGF-1 levels.

### Neuropsychological index

Participants in the control group had significantly lower accuracy rates in the oddball condition after 12 months than at baseline, indicating that older adults exhibit a reduced ability with aging to differentiate between standard and target stimuli during the three-stimulus oddball task. The results of the present study support those of previous research, as healthy individuals aged 55–80 years showed a decrease of 0.21 in mean MMSE score per year, with 22% of individuals showing a decrease of more than 1 point per year (Kalmijn et al., 2000), and elderly individuals demonstrated lower accuracy for novel stimuli (Fabiani and Friedman, 1995). These findings demonstrate that aging is marked by a progressive decline in cognitive functioning, and that information processing is vulnerable to aging. However, the participants in the exercise group did not exhibit a similar trend of decreased performance during the study intervention period, suggesting that these negative effects of aging may be attenuated by regular participation in resistance exercise.

In addition, the exercise group displayed faster RTs in the oddball and novel conditions after the exercise intervention compared with at baseline, and, in particular, this change led to a group difference in the oddball condition after 12 months. These findings suggest that the neuromotor and central processing of cognitive functions used to distinguish the oddball stimulus from a frequent stimulus could be significantly enhanced in elderly males by 12 months of resistance exercise. Indeed, Hatta et al. (2005) found that regular participation in moderate exercise could promote response processing when performing a somatosensory oddball task in elderly adults. In addition, a previous study demonstrated that elderly individuals who are physically active could retain their reaction capacity (Spirduso, 1980). Based on findings from both the present study and previous research, we postulate that high-intensity resistance exercise could facilitate greater temporal efficiency in the central processing of cognitive functions in healthy elderly individuals.

Interestingly, the group differences seen in task accuracy and RT were driven by different patterns of behavioral changes during a one-year period. That is, the accuracy in the oddball condition declined significantly in the control group, while the RT decreased significantly in the exercise group. Previous studies examined the accuracy rates and RTs during completion of an oddball task among subjects of various ages, and found that elderly adults are less accurate relative to middle-aged and younger adults, whereas no statistically significant differences in RTs were observed, showing that aging has different effects on these two outcomes, with the authors suggesting that this may be due to age-related changes in response strategies (Ford and Pfefferbaum, 1985; Iragui et al., 1993). In this study we observed that, relative to baseline performance, the control group exhibited decreased accuracy with maintenance of RT performance. This is probably because the elderly adults in the control group adopted a strategy that favors processing speed over accuracy when performing this type of cognitive task, which diminishes RT delays at the expense of decreased accuracy. Similarly, for the exercise group, the benefits of exercise training were easier to observe with regard to RTs, since these subjects also focused more on speed than accuracy, and this trade-off may thus reduce any benefits with regard to task accuracy.

### Neuroelectric index

P300 reflects attentional processes, indexed by two distinct yet related subcomponents of neural processes (Pontifex et al., 2009): the P3a component is elicited by a change in the stimulus environment (e.g., an infrequent or novel non-repeating distractor), with the P3a amplitude reflecting a stimulus-driven, or bottom-up, attentional orienting to a salient but irrelevant stimulus (Polich, 2007; Richardson et al., 2011); the P3b component is elicited by a rare stimulus within a series of frequent irrelevant stimuli (O’Connell et al., 2012), with the P3b amplitude serving as a proposed reflection of the top-down allocation of attentional resources to stimulus evaluation when working memory is updated (Polich, 2007; Verleger, 2008). In the present study, the control group exhibited significantly smaller P3a and P3b amplitudes in the oddball condition after 12 months, indicating age-related decreases in both attentional orienting/engagement of focal attention, and attentional resource allocation and subsequent memory processing (Polich, 2007; Verleger, 2008) in healthy elderly subjects. These findings echo those of a previous study that examined the extent of the decline in attention control efficiency during normal aging, and which suggested that reduced attention control in older adults relative to younger adults, observed in terms of cortical activity, causes greater inefficiency in the tendency to filter out irrelevant information over successive trials (Fabiani et al., 2006).

In contrast, the exercise group sustained P3 amplitudes in the oddball condition over 12 months of high-intensity resistance exercise, resulting in a significant difference in P3 amplitudes between the exercise and control groups. Similarly, Polich and Lardon (1997) demonstrated that very physically active young adults achieved larger P3 amplitudes compared to relatively inactive counterparts when performing the visual oddball task. However, Pontifex et al. (2009) examined P300 components separately, and found that older adults with high cardiorespiratory fitness only exhibited greater P3b amplitudes, with no significant differences in P3a amplitudes relative to controls, suggesting that fitness-related changes in cognitive aging appear specific to attentional processing. In the present study, the exercise group exhibited significantly larger P3a and P3b amplitudes during the stimulus discrimination task, indicating that 12 months of resistance training can simultaneously maintain the capacities for both orienting and allocating attention. The present neuroelectric findings seem to suggest that chronic resistance exercise might modulate, and in some cases, potentially reverse, age-related decreases in neuronal tissue loss in the brain cortices.

### Neurophysiological index

Although GH stimulation can cause an increase in IGF-1 production (Sonntag et al., 2005), the healthy elderly males in the current study showed significant increases in basal serum IGF-1 levels after 12 months of high-intensity resistance exercise, without any accompanying elevation in GH levels from baseline. This result supports previous studies demonstrating that spontaneous GH secretion does not positively correlate with basal IGF-1 levels in older adults (Vermeulen, 1987; Benbassat et al., 1997). In this study, the slight increase in GH levels in the exercise group after long-term exercise does not indicate a significant relationship with changes in neuropsychological and neuroelectric performance. This substantiates previous studies that investigated neurocognitive performance in subjects with GH replacement. For example, Papadakis et al. (1996) found that 6 months of GH treatment did not result in a significant improvement in neuropsychological performance in healthy elderly men with low baseline IGF-1 levels. Golgeli et al. (2004) also reported that 6 months of GH replacement therapy in Sheehan syndrome patients with severe GH deficiency did not significantly affect P3 amplitudes. More recently, Quik et al. (2012) failed to find a relationship between GH levels and N2b amplitudes in healthy males aged 50–78 years during performance of a selection-potential go/no-go task. However, Rollero et al. (1998) observed that although the MMSE score was not associated with basal GH levels or GH peaks after GH-releasing hormone stimulation in elderly subjects, cognitive performance was positively related with total IGF-1 levels.

Despite a decrease in circulating serum IGF-1 levels paralleling a decline in GH pulses in later life (Corpas et al., 1993), the close relationship between them seems not to extend to identical effects on cognitive performance. Indeed, only changes in the IGF-1 levels of the exercise group subjects in the current study correlated significantly with changes in RTs and P3b amplitudes, with such an effect not exhibited by the GH parameter. Previous studies have demonstrated that age-related decreases in serum IGF-1 levels could be a potential mechanism for age-related decline in cognitive functions (e.g., processing speed) in the elderly (Sonntag et al., 2005). However, increased brain uptake of peripheral IGF-1 during exercise could lead to training-induced neuroprotective effects (Carro et al., 2000). That is, IGF-1 is essential for exercise-induced neurogenesis (Carro et al., 2000), and acts to mediate exercise-induced angiogenesis (Lopez-Lopez et al., 2004). In the current study, IGF-1 levels were significantly increased in the exercise group after 12 months of resistance exercise training. The changes in IGF-1 levels significantly correlated with RT performance, reflecting previous research which demonstrated that higher IGF-1 levels in adults with Prader–Willi syndrome are associated with faster temporal memory performance (van Nieuwpoort et al., 2011). The findings of the present study show that increases in IGF-1 levels after long-term resistance exercise can reduce the time needed for central processing of cognitive functions (e.g., RTs) in healthy elderly males. Similarly, Baker et al. (2010) found that aerobic fitness may improve executive control with an increase in IGF-1 in elderly males at risk of cognitive disorder. However, although Aleman et al. (1999) reported no correlation between IGF-1 levels and memory, attention, or fluid intelligence in healthy elderly males aged 65–76 years, they did observe significant associations for serum IGF-1 levels with both perceptual–motor and information processing speed, which is known to decline significantly with aging. Based on our findings and those of previous studies, serum IGF-1 levels could be the key neurophysiological indicator of improved response processing in healthy elderly males after participation in 12 months of high-intensity resistance exercise. However, such a positive effect did not appear to emerge in the neural system during the aging process, and thus increased serum IGF-1 levels do not increase P3a and P3b amplitudes in healthy elderly males.

The current study found a significant negative correlation between the changes in IGF-1 levels and P3b amplitudes for the exercise group subjects in the oddball condition, suggesting that enhanced IGF-1 levels might have decreased the neural system degeneration, and thus enabled them to better distinguish the oddball stimulus from the standard stimulus. However, the changes in IGF-1 levels were only associated with P3b amplitude, not P3a amplitude, indicating that changes in the growth factor were not related to a general change in the attentional system. Serum IGF-1 levels appear to selectively associate with a particular aspect of attention; specifically, IGF-1 might mediate the neural network involved in the top-down allocation of attentional resources, while not affecting the bottom-up allocation used during attentional orienting. Since P3a and P3b might relate to the dopaminergic and locus-coeruleus-norepinephrine systems, respectively (Nieuwenhuis et al., 2005; Polich and Criado, 2006), further research is warranted in this area, with a possible focus on examining the potential interactive mechanisms between IGF-1 and these two biochemical systems.

Overall, the evidence presented above suggests that IGF-1 might be an intermediary for the effects of resistance exercise at central levels, despite previous studies reporting that the upregulation of the GH/IGF-1 axis seemed to produce positive effects on cognitive functions. Indeed, Kalmijn et al. (2000) found that higher total serum IGF-1 concentrations at baseline significantly correlated with less cognitive decline in terms of MMSE score over a two-year period in healthy individuals aged 55–88 years. Serum IGF-1 concentrations might reflect an underlying biological process influencing cognitive decline based on the statistically significant correlations obtained between changes in IGF-1 levels and cognitive performance. Collectively, these results indicate that this biochemical agent can attenuate or minimize the progress of certain aspects of cognitive degeneration in healthy elderly individuals. This might be achieved through various central mechanisms, including actions on neurons, cerebrovasculature, and the number of cells expressing c-fos in neurons and glia, possibly because of its transportation to the central nervous system via the hematoencephalic barrier (Sonntag et al., 2005). Additionally, these findings indicate that factors other than GH secretion are involved in the relationship between serum IGF-1 levels and cognitive function decline in the elderly.

High homocysteine levels are a risk factor for cognitive impairment in older adults (Ford et al., 2012). In the present study, serum homocysteine levels were significantly reduced in the exercise group after 12 months of resistance exercise, echoing the findings of a previous study in which serum homocysteine decreased after 6 months of high- or low-intensity resistance exercise (Vincent et al., 2003). Although the potential mechanisms by which resistance training might prevent cognitive decline in the elderly involve homocysteine (Liu-Ambrose and Donaldson, 2009), the present study did not show an association between changes in homocysteine levels and changes in neuropsychological and neuroelectric measures. A possible explanation for this is that the cognitive task adopted in this study related to executive functions, with new research suggesting that high homocysteine levels in elderly adults only decrease performance in tests of immediate and delayed memory, not executive functions (Ford et al., 2013). Although a few studies have demonstrated prolonged potential latencies in P3 amplitude associated with elevated homocysteine levels (Evers et al., 1997; Díaz-Leines et al., 2013), as in all experimental studies with a cross-sectional design, it is difficult to infer causal relationships between serum homocysteine levels and neurocognitive performance in healthy elderly males.

### Study limitations

While the present findings shed light on the beneficial effects of 12 months of high intensity resistance training on neuropsychological, neuroelectric, and neurophysiological outcomes, there are some limitations that indicate they should be applied with caution. First, we only recruited male elderly adults in the present study to exclude the influences of gender differences in executive control (Rubia et al., 2010), myofiber hypertrophy (Bamman et al., 2003) and endocrine indices (Staron et al., 1994) in response to resistance training. The results may thus not be generalized to female subjects without more work being done. Second, the results of the present experimental design (i.e., a single laboratory-based cognitive task) might be difficult to apply to daily living activities. An additional virtual reality task (i.e., Chaddock et al., 2012) may thus help to assess the potential behavioral benefits of resistance exercise in future investigations.

## Conclusions

In conclusion, increasing the level of physical activity via high-intensity resistance exercise could assist in lowering the rate of age-related neurocognitive decreases in healthy elderly males. In addition, increases in basal IGF-1 levels achieved via such an exercise protocol could have positive effects on both neuropsychological (i.e., RT) and neuroelectric (i.e., P3b amplitude) performance in the elderly. This study’s findings imply that healthy elderly individuals who regularly engage in resistance exercise might delay the onset of age-related decline in executive functions, and that this protective effect may be modulated by the growth factor-IGF-1.

## Conflict of interest statement

The authors declare that the research was conducted in the absence of any commercial or financial relationships that could be construed as a potential conflict of interest.
